# A case of severe side effect due to muscle oil injections in combination with chemotherapy in a former bodybuilder

**DOI:** 10.1002/ccr3.4444

**Published:** 2021-07-23

**Authors:** Gro Gitz‐Johansen, Anne Birgitte Als, Kristine Appel Uldall Pallesen

**Affiliations:** ^1^ Department of Dermatology Aarhus University Hospital Aarhus N Denmark; ^2^ Department of Oncology Aarhus University Hospital Aarhus N Denmark

**Keywords:** bodybuilder, chemotherapy, oil injections, site‐enhancing oils, skin reaction

## Abstract

This case report presents an acute painful skin reaction several years after injection of site‐enhancing oils (SEO) induced by chemotherapy. This case exemplifies the long‐term dangerous side effects of the use of SEO, however treatable with prednisolone.

## Graphical abstract

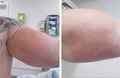

## 1 INTRODUCTION

Site‐enhancing oils (SEO) injections were introduced in 1899 for breast enlargement.^1^ The procedure is still used in the bodybuilder subculture to enhance appearance of the muscles. The complications have earlier been documented as ulcerations, multiple painful nodules, and muscle deformity with fibrosis.^2^, ^3^, ^4^ We present a 30‐year‐old former bodybuilder treated with chemotherapy due to seminoma testicular cancer with swelling of the muscles who were prior treated with SEO injections.

## 2 CASE

A 30‐year‐old man known with chemotherapy‐treated seminoma testicular cancer, phenylketonuria, and paraffin oil‐induced hypercalcemia was admitted to the hospital due to 7 days of suspected erysipelas and fever.

The patient was diagnosed with retroperitoneal lymph node metastases 6 months after orchiectomy. He was treated with cisplatin, etoposide, and bleomycin according to Danish national guidelines. Fourteen days after the first dose of chemotherapy, the patient had an abscess caused by a venous access port. This was treated by incision and was initially recovering, but 2 days later the patient was readmitted with high temperature.

**FIGURE 1:**
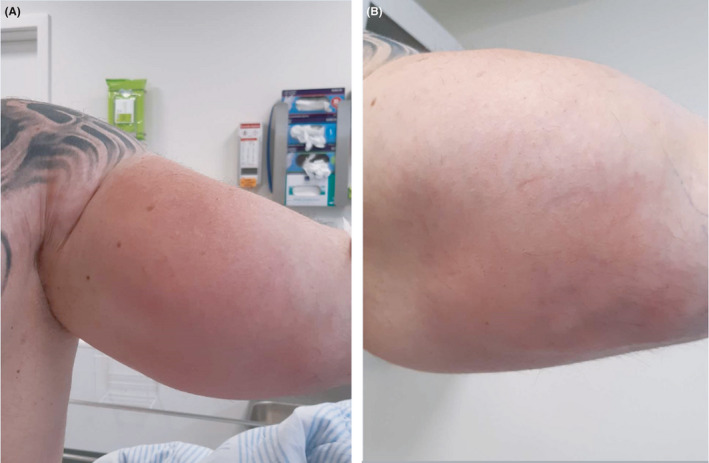
To the left (A): left upper arm prior to treatment. Patient presents with swelling of upper arm with severe inflammation of the skin. To the right (B): Left upper arm 4 days after treatment with prednisolone

Cutaneous examination showed severe inflamed skin and expanded painful muscle groups related to the shoulders, upper arms, and torso (Figure 1A). The peripheral blood sample showed CRP of 299 mg/L [<8.0] with normal leukocytes. The body temperature was measured to 40 ˚C. He was initially treated with intravenous injections of piperacillin/tazobactam and meropenem, which reduced CRP to 170 mg/L [<8.0], but the body temperature was still 39–40°C and no improvement of the skin and muscle symptoms was seen. Several blood culture samples were done, all negative. Magnetic resonance imaging (MRI) of the right upper arm showed muscular edema and several myriads of lipid drops in the muscles compatible to oil inclusions. A daily dose of 50 mg oral prednisolone was added for 5 days with an improvement in cutaneous and muscular symptoms and additionally CRP reductions to 94 mg/L [<8.0] (Figure 1B).

Due to this severe reaction and the seminoma pathology, the chemotherapy was disrupted, and the patient received external beam radiotherapy according to guidelines and was in complete remission at his 3‐month follow‐up visit.

Eight‐ to 10‐year prior admission, the patient was treated by a friend from the bodybuilder environment with intramuscular injection of paraffin and synthol. He injected 50 mL at a time into several muscle groups. The treatment was given every 3 months over a 2‐year period with the last treatment 3 years ago.

## 3 DISCUSSION AND CONCLUSION

Site‐enhancing oils injections used in the bodybuilder subculture have in recent years become a growing issue.^5^ SEO injections often consist of a mixture of lidocaine, alcohol, and an oil substrate.

Diagnosis typically involves MRI showing oil inclusions and/or biopsy from the involved muscle, and diagnosis often relies on honesty in anamnesis from the patient.

Treatment depends on the specific case severity ranging from surgical procedures, steroids, antibiotics, and compression therapy.^2^

Site‐enhancing oils injections provoke biologically responses in different stages. First, an acute inflammatory response followed by a latent phase in which the oil can be well tolerated. The latent phase can last several years, like in this particular case. Depending on the patient's fat tissue and the amount of injected oil, the fatty acids will over time discharge and combine with calcium to form hyaline sclerosis in the last phase.^1^ This is due to macrophage activation and will over time cause, as previously described, nodules, ulcerations, and atypical skin reactions.

This case is interesting in which the skin reaction appears after chemotherapy. It can be argued that a chemical reaction from the chemotherapy in this case drew the response from oil injection from a latent phase to a reactive phase in which an immune response was provoked.

In this case, the patient showed hypercalcemia, which previously has been described as a known side effect to SEO injections, which could potentially lead to severe kidney failure.^7^

Erysipelas was a differential diagnosis, and as seen in Figure 1, the skin reaction has clinical similarities to erysipelas. It could have led to wrong diagnosis and treatment with antibiotics without result and maybe worsening of the painful reaction, longer treatment time, higher doses of prednisolone, and misuse of antibiotics contributing to resistance.

Unfortunately, the bodybuilder community is misinformed and mislead by the information on SEO provided from the internet, which Petersen et al.^4^ highlights as problematic as well.

As argued by Henriksen et al.,^6^ resources should be put in order to prevent use and misuse of SEO in the bodybuilder community, for example, in form of medical education by doctors in the community.

## CONFLICT OF INTEREST

None declared.

## AUTHOR CONTRIBUTIONS

All authors provided consent for the publication of this manuscript. All authors have contributed to the work according to all four criteria listed under authorship in author guidelines.

## Data Availability

Data Availability Statement Data regarding this case report can be accessed by contacting corresponding author.
